# Association of D-dimer Levels with Clinical Event Rates and the Efficacy of Betrixaban versus Enoxaparin in the APEX Trial

**DOI:** 10.1055/s-0037-1615288

**Published:** 2018-01-08

**Authors:** C. Michael Gibson, Lisa K. Jennings, Gerald Chi, Megan K. Yee, Rim Halaby, Tarek Nafee, Fahad AlKhalfan, Mathieu Kerneis, Serge Korjian, Yazan Daaboul, Samuel Z. Goldhaber, Russel D. Hull, Adrian F. Hernandez, Alexander T. Cohen, Robert A. Harrington

**Affiliations:** 1 Cardiovascular Division, Department of Medicine, Beth Israel Deaconess Medical Center, Harvard Medical School, Boston, Massachusetts, United States; 2 CirQuest Labs, The University of Tennessee Health Science Center, Memphis, Tennessee, United States; 3 Cardiovascular Division, Brigham and Women's Hospital, Harvard Medical School, Boston, Massachusetts, United States; 4 Division of Cardiology, Faculty of Medicine, University of Calgary, Alberta, Canada; 5 Division of Cardiology, Duke University and Duke Clinical Research Institute, Durham, North Carolina, United States; 6 Department of Haematological Medicine, Guy's and St. Thomas' Hospitals, King's College London, London, United Kingdom; 7 Department of Medicine, Stanford University, Stanford, California, United States

**Keywords:** D-dimer, betrixaban, deep vein thrombosis, pulmonary embolism, venous thromboembolism

## Abstract

**Background** Elevated D-dimer concentrations are associated with an increased risk of venous thromboembolism (VTE). However, they may also provide prognostic value. The present analysis sought to study the association of D-dimer levels with VTE event rates and the efficacy of betrixaban versus enoxaparin in the APEX trial.

**Methods** Hospitalized acutely medically ill subjects ( *n* = 7,513) were randomized in a double-dummy double-blind fashion to either extended-duration oral betrixaban (80 mg once daily for 35–42 days) or standard dose subcutaneous enoxaparin (40 mg once daily for 10 ± 4 days) for venous thromboprophylaxis. D-dimer was assessed using a central core laboratory measurement.

**Results** For every 0.25 µg/mL increase in D-dimer concentration, there was a 2% increase in the relative risk of experiencing the primary efficacy endpoint (asymptomatic deep vein thrombosis [DVT], symptomatic DVT, nonfatal pulmonary embolism, or VTE-related death) in both the betrixaban ( *p* < 0.001) and enoxaparin ( *p* < 0.001) treatment arms. Among D-dimer-positive (≥ 2 × upper limit of normal; corresponding to ≥ 1.00 µg/mL) subjects, extended-duration betrixaban reduced the risk of experiencing the primary efficacy outcome (5.4% [ *n* = 124] vs. 7.6% [ *n* = 170]; odds ratio = 0.69; 95% confidence interval: 0.55–0.88; absolute risk reduction = 2.2%, number needed to treat = 46, *p* = 0.003). There was no interaction between D-dimer and the treatment effect ( *p*_int_ = 0.53).

**Conclusion** Extended-duration betrixaban was superior to standard-duration enoxaparin, irrespective of D-dimer level at baseline. To prevent one VTE event, 46 D-dimer-positive patients would need to be treated with betrixaban.

## Background

D-dimer is a fibrin degradation product and a marker for clot formation and lysis. D-dimer is elevated in a wide variety of illnesses, most notably venous thromboembolism (VTE), 1 2 3 and is used as a diagnostic screening tool to exclude VTE among subjects with a low-to-intermediate clinical probability of deep vein thrombosis (DVT) or pulmonary embolism (PE). 4 5 6 Elevated D-dimer levels are also associated with an increased risk of recurrent VTE and mortality. 7 8 9 10 Since D-dimer concentration is often used to identify medical subjects at a high risk of VTE, we hypothesized that D-dimer may help identify subjects who might have a modifiable risk and who might benefit from extended-duration thromboprophylaxis following hospitalization for an acute medical illness. The aims of this analysis were to evaluate the association of D-dimer with VTE outcomes and to assess possible modulation of the treatment effect of betrixaban versus enoxaparin as a function of D-dimer concentration.

## Methods

### Study Design and Endpoints

The Acute Medically Ill VTE Prevention with Extended Duration Betrixaban (APEX) trial was a randomized, double-blind, double-dummy, active controlled, multinational clinical trial that evaluated the efficacy and safety of extended-duration VTE prophylaxis (ClinicalTrials.gov identifier: NCT01583218). Acutely ill hospitalized medical subjects at risk of VTE ( *n* = 7,513) were randomized to standard-duration enoxaparin for 10 ± 4 days ( *n* = 3,754) or extended-duration betrixaban for 35 to 42 days ( *n* = 3,759). The study design and primary results of the APEX trial have been previously published. 11 12

The primary efficacy outcome was the composite of asymptomatic proximal DVT, symptomatic proximal or distal DVT, symptomatic nonfatal PE, or death from VTE through visit 3 or day 42. The primary efficacy outcome was also assessed through the end of the study or day 77. The primary safety endpoint of the study was major bleeding through 7 days after discontinuation of study medication. D-dimer levels were measured at the time of enrollment by both central (CirQuest Labs, Memphis, Tennessee, United States) and local laboratories. Quantitative D-dimer methods employed at local sites include the turbidimetric assay, enzyme-linked immunosorbent assay, and STA Liatest D-Di immunoturbidimetric assay (Diagnostica Stago, Asnières-sur-Seine, France). At the central laboratory, D-dimer was measured by the STA Liatest D-Di immunoturbidimetric assay (Diagnostica Stago, Asnières-sur-Seine, France).

### Statistical Analysis

Analyses were performed using SAS version 9.4 (Cary, North Carolina, United States). In this substudy, a positive D-dimer was defined as ≥ 2 × upper limit of normal (ULN) (corresponding to ≥ 1.00 µg/mL) as measured by the central laboratory. If the central D-dimer was missing, the value was imputed using the local laboratory measurement. D-dimer was analyzed as both a categorical variable (positive [≥ 2 × ULN] or negative [< 2 × ULN]) and as a continuous variable. Analyses were further stratified by dosing criteria (severe renal dysfunction or strong concomitant P-glycoprotein inhibitor use, or neither) within the D-dimer-positive and D-dimer-negative groups.

Baseline characteristics were compared across D-dimer tertiles (T _1_ : < 0.85 µg/mL; T _2_ : ≥ 0.85 to < 1.89 µg/mL; T _3_ : ≥ 1.89 µg/mL) and stratified by treatment arms. Values for continuous variables were reported as the mean ± standard deviation or median and interquartile range when specified. Values for categorical variables were reported as the number and proportion. A two-sample *t* -test was used to evaluate the difference in means for continuous variables between treatment groups within each tertile. Analysis of variance and Kruskal–Wallis tests (if median reported) were used to test the difference of means across tertiles. A chi-square test was used for the analysis of categorical variables.

Efficacy was assessed in the modified intention-to-treat (mITT) population, which consists of all subjects who were administered at least one dose of study drug and in whom follow-up assessment data were evaluable on one or more efficacy components. A comparison to the MAGELLAN trial was performed to match the methodology used in that study, using only central D-dimer (not imputing local D-dimer if central is missing) and the primary efficacy outcome population, which includes all patients in the mITT population who had assessment of all components of the primary efficacy endpoint. All efficacy analyses used treatment as stratified. The safety endpoints were analyzed within the safety population, which included all randomized subjects who received any portion of either study drug. All safety analyses used treatment as received.

The risk of VTE was modeled using D-dimer concentration in a logistic regression model, within each treatment arm. Logistic regression was used to determine the increase in odds of VTE comparing betrixaban to enoxaparin stratified by D-dimer category. The independent relationship between D-dimer and VTE was assessed by fitting a multivariate model. To control for possible confounders of VTE, a stepwise selection approach at a significance threshold of *p* < 0.15 was applied. The covariates were retained in the final model if the adjusted *p* -value was < 0.05, which included age, duration of hospitalization, prior VTE, and previous thromboprophylaxis ≤ 96 hour. Finally, the interaction term of D-dimer (as both a categorical and continuous variable) on the treatment effect was tested.

## Results

### Baseline Characteristics

Baseline characteristics were well-balanced across D-dimer tertiles and between treatment arms ( Table 1 ). There were three subjects in the betrixaban arm and four subjects in the enoxaparin arm that were missing both central and local laboratory measurements.

**Table 1:** Characteristics of subjects at baseline

	1st tertile			2nd tertile			3rd tertile			
Characteristic a	Enoxaparin, *N* = 1,233	Betrixaban, *N* = 1,238	*p* -Value	Enoxaparin, *N* = 1,241	Betrixaban, *N* = 1,235	*p* -Value	Enoxaparin, *N* = 1,240	Betrixaban, *N* = 1,240	*p* -Value	6-way *p* -value
Age, y	76.0 ± 8.12	76.3 ± 7.97	0.46	76.6 ± 8.42	77.1 ± 8.64	0.14	75.9 ± 8.39	76.5 ± 8.55	0.10	**0.008**
Male sex, no. (%)	562 (45.6)	583 (47.1)	0.45	555 (44.7)	553 (44.8)	0.98	581 (46.9)	554 (44.7)	0.28	0.693
Mean weight, kg	82.4 ± 19.70	81.1 ± 19.50	0.11	80.2 ± 19.74	80.4 ± 19.50	0.80	79.6 ± 18.61	78.1 ± 18.54	**0.04**	**<0.001**
Body mass index b	30.2 ± 6.73	29.7 ± 6.80	0.06	29.5 ± 6.99	29.4 ± 6.75	0.81	28.9 ± 6.27	28.5 ± 6.20	0.10	**<0.001**
Median no. of days hospitalization (IQR)	10 (8–14)	10 (7–14)	0.73	10 (8–14)	10 (7–14)	0.44	10 (8–14)	10 (7–14)	0.17	0.20
Creatinine clearance, no. (%) c			0.42			0.95			0.09	**<0.001**
< 15 mL/min	0	0	–	0	0	–	0	1 (0.1)	–	–
15 to <30 mL/min	30 (2.4)	39 (3.2)	–	52 (4.2)	50 (4.1)	–	67 (5.4)	84 (6.8)	–	–
30 to <60 mL/min	473 (38.4)	486 (39.3)	–	520 (41.9)	526 (42.6)	–	523 (42.2)	569 (45.9)	–	–
60 to <90 mL/min	459 (37.2)	461 (37.2)	–	443 (35.7)	436 (35.3)	–	433 (34.9)	388 (31.3)	–	–
≥90 mL/min	269 (21.8)	252 (20.4)	–	223 (18.0)	218 (17.7)	–	214 (17.3)	192 (15.5)	–	–
Missing	2 (0.2)	0 (0.0)	–	3 (0.2)	5 (0.4)	–	3 (0.2)	6 (0.5)	–	–
Race or ethnic group, no. (%) d			0.07			0.10			0.59	**0.02**
White	1175 (95.3)	1174 (94.9)	–	1156 (93.2)	1146 (92.8)	–	1155 (93.2)	1144 (92.3)	–	–
Asian	3 (0.2)	3 (0.2)	–	1 (0.1)	4 (0.3)	–	3 (0.2)	2 (0.2)	–	–
Black	23 (1.9)	12 (1.0)	–	19 (1.5)	32 (2.6)	–	28 (2.2)	26 (2.1)	–	–
Other	32 (2.6)	49 (4.0)	–	65 (5.2)	53 (4.3)	–	54 (4.4)	68 (5.5)	–	–
Concomitant P-glycoprotein inhibitor, no. (%)	216 (17.5)	223 (18.0)	0.75	190 (15.3)	208 (16.8)	0.30	238 (19.2)	238 (19.2)	>0.999	0.09
Previous thromboprophylaxis ≤ 96 h, no. (%)	585 (47.5)	639 (51.6)	**0.04**	647 (52.1)	650 (52.6)	0.80	621 (50.1)	613 (49.4)	0.75	0.20
Acute medical condition, no. (%)			0.98			0.48			0.78	**<0.001**
Heart failure	568 (46.1)	579 (46.8)	–	541 (43.6)	545 (44.1)	–	555 (44.8)	546 (44.0)	–	–
Infection	246 (20.0)	247 (20.0)	–	359 (28.9)	387 (31.4)	–	435 (35.1)	459 (37.0)	–	–
Respiratory failure	203 (16.5)	194 (15.7)	–	149 (12.0)	131 (10.6)	–	113 (9.1)	114 (9.2)	–	–
Ischemic stroke	177 (14.4)	180 (14.5)	–	153 (12.3)	134 (10.9)	–	99 (8.0)	89 (7.2)	–	–
Rheumatic disorder	39 (3.2)	36 (3.0)	–	39 (3.1)	38 (3.1)	–	38 (3.1)	32 (2.6)	–	–
Risk factor for venous thromboembolism, no. (%)										
Age ≥75 y	862 (69.9)	890 (71.9)	0.28	852 (68.7)	858 (69.5)	0.66	775 (62.5)	801 (64.6)	0.28	**<0.001**
History of cancer	159 (12.9)	177 (14.3)	0.31	144 (11.6)	129 (10.5)	0.36	134 (10.8)	152 (12.3)	0.26	**0.04**
History of deep-vein thrombosis or pulmonary embolism	98 (8.0)	97 (7.8)	0.92	74 (6.0)	110 (8.9)	**0.005**	118 (9.5)	98 (7.9)	0.15	**0.031**
History of New York Heart Association class III or IV heart failure	290 (23.5)	284 (22.9)	0.73	299 (24.1)	299 (24.2)	0.95	270 (21.8)	265 (21.4)	0.81	0.42
Concurrent acute infectious disease	197 (16.0)	173 (14.0)	0.16	204 (16.4)	211 (17.1)	0.67	210 (16.9)	212 (17.1)	0.92	0.26
Severe varicosities	267 (21.7)	256 (20.7)	0.55	209 (16.8)	241 (19.5)	0.08	207 (16.7)	200 (16.1)	0.70	**<0.001**
Hormone-replacement therapy	11 (0.9)	9 (0.7)	0.65	10 (0.8)	17 (1.5)	0.17	10 (0.8)	16 (1.3)	0.24	0.43
Known thrombophilia e	0	2 (0.2)	0.50	3 (0.2)	1 (0.1)	0.62	2 (0.2)	0	0.50	0.42

Significant results ( *p* -value < 0.05) are expressed in bold.

Note: D-dimer tertiles: T _1_ : < 0.85 µg/mL; T _2_ : ≥ 0.85 to < 1.89 µg/mL; T _3_ : ≥ 1.89 µg/mL.

a Plus–minus values are ± SD. IQR denotes interquartile range.

b The body mass index is the weight in kilograms divided by the square of the height in meters.

c Creatinine clearance levels were calculated with the use of the Cockcroft-gault equation on the basis of creatinine levels on day 1. To convert the values for creatinine to micromolecules per liter, multiply by 88.4.

d Race or ethnic group was self-reported. “Other” includes subjects who were categorized as Native American, Alaska Native, Native Hawaiian or Pacific Islander, other race, or mixed.

e Defined as inherited or acquired disorder of hemostasis including antithrombin III deficiency, protein C deficiency, and protein S deficiency.

### D-dimer and Efficacy Outcomes

For every 0.25 µg/mL unit increase in D-dimer concentration, there was a significant increase in the relative risk of a VTE event (odds ratio [OR] = 1.02; 95% confidence interval [CI]: 1.01–1.03; *p* < 0.001). This was true in both the betrixaban (OR = 1.02 [95% CI: 1.01–1.03]; *p* < 0.001) and enoxaparin (OR = 1.02 [95% CI: 1.01–1.03]; *p* < 0.001) treatment arms. In a multivariate analysis adjusting for other variables associated with the primary endpoint, D-dimer concentration as a continuous variable remained independently associated with VTE events (OR = 1.02 [95% CI: 1.01–1.03]; *p* < 0.001; Table 2 ). There was no significant interaction between D-dimer concentration as a continuous variable and the treatment effect ( *p*_int_ = 0.87; Table 3 ).

**Table 2:** Multivariate analysis of D-dimer concentration and D-dimer category modeling the primary efficacy outcome through visit 3

Covariate	Univariate model		Multivariate model for D-dimer concentration a		Multivariate model for D-dimer category a	
	OR (95% CI)	*p* -Value	Adjusted OR (95% CI)	*p* -Value	Adjusted OR (95% CI)	*p* -Value
D-dimer concentration (per 0.25 µg/mL increase)	1.02 (1.01–1.03)	**<0.001**	1.02 (1.01–1.03)	**<0.001**		
D-dimer category ≥ 2× ULN vs. < 2× ULN	2.10 (1.66–2.67)	**<0.001**			2.03 (1.59–2.58)	**<0.001**
**Baseline characteristics**						
Age, y	1.01 (0.99–1.02)	0.146	1.02 (1.01–1.03)	**0.002**	1.02 (1.01–1.03)	**0.002**
Sex—male vs. female	1.07 (0.87–1.31)	0.521	–	–	–	–
Mean weight, kg	0.99 (0.99–1.00)	0.254	–	–	–	–
Body mass index	0.99 (0.97–1.00)	0.124	–	–	–	–
Duration of hospitalization, d	1.03 (1.02–1.04)	**<0.001**	1.03 (1.01–1.04)	**<0.001**	1.02 (1.01–1.04)	**0.0002**
Creatinine clearance		0.803				
< 15 mL/min b	–	–	–	–	–	–
15 through < 30 mL/min	1.29 (0.81–2.04)	0.281	–	–	–	–
30 through <60 mL/min	REF	REF	–	–	–	–
60 through < 90 mL/min	0.92 (0.73–1.17)	0.499	–	–	–	–
≥ 90 mL/min	0.92 (0.69–1.23)	0.557	–	–	–	–
Missing b	–	–	–	–	–	–
Race		0.412	–	–	–	–
White	REF	REF	–	–	–	–
Black	1.41 (0.73–2.70)	0.305	–	–	–	–
Asian	2.62 (0.59–11.55)	0.204	–	–	–	–
Other	0.89 (0.53–1.51)	0.668	–	–	–	–
Concomitant P-glycoprotein inhibitor—Y vs. N	0.95 (0.73–1.25)	0.731	–	–	–	–
Previous thromboprophylaxis ≤ 96 h—Y vs. N	1.34 (1.09–1,65)	**0.005**	1.36 (1.10–1.68)	**0.005**	1.33 (1.08–1.64)	**0.008**
Admission criteria		0.206				
Heart failure	REF	REF	–	–	–	–
Infection	1.30 (0.95–1.80)	0.106	–	–	–	–
Respiratory failure	1.23 (0.96–1.57)	0.106	–	–	–	–
Ischemic stroke	1.41 (0.80–2.48)	0.232	–	–	–	–
Rheumatic disorder	1.36 (0.98–1.88)	0.066	–	–	–	–
History of cancer—Yes vs. No	1.25 (0.93–1.67)	0.138	–	–	–	–
History of deep-vein thrombosis or pulmonary embolism—Yes vs. No	3.70 (2.87–4.78)	**<0.001**	3.92 (2.99–5.12)	**<0.001**	3.85 (2.94–5.04)	**<0.001**
History of New York Heart Association class III or IV heart failure—Yes vs. No	0.98 (0.77–1.26)	0.896	–	–	–	–
Concurrent acute infectious disease—Yes vs. No	1.06 (0.81–1.39)	0.665	–	–	–	–
Severe varicosities—Yes vs. No	0.98 (0.75–1.28)	0.882	–	–	–	–
Hormone replacement therapy—Yes vs. No	1.64 (0.71–3.80)	0.251	–	–	–	–
Known thrombophilia—Yes vs. No c	6.10 (1.23–30.29)	**0.027**	–	–	–	–

Significant results ( *p* -value < 0.05) are expressed in bold.

Abbreviations: OR, odds ratio; ULN, upper limit of normal; VTE, venous thromboembolism.

a Independent baseline characteristics were included in a stepwise regression model if univariate *p* < 0.15 and were retained in the final model if adjusted *p* < 0.05.

b Not enough data to produce estimate.

c Defined as inherited or acquired disorder of hemostasis including antithrombin III deficiency, protein C deficiency, and protein S deficiency.

**Table 3:** Interaction of D-dimer category or D-dimer concentration and treatment effect through visit 3

Outcome a	Enoxaparin	Betrixaban	Odds ratio (95% CI)	*p* -Value b	*p* -Value for interaction c	*p* -Value for interaction d
Primary efficacy outcome						
≥2 × ULN *n* / *N* (%)	170/2,236 (7.6)	124/2,291 (5.4)	0.69 (0.55–0.88)	**0.003**	0.53	0.87
<2 × ULN *n* / *N* (%)	52/1,480 (3.5)	41/1,427 (2.9)	0.82 (0.54–1.24)	0.33		

Significant results ( *p* -value < 0.05) are expressed in bold.

a The primary efficacy outcome is a composite of asymptomatic proximal DVT, symptomatic proximal or distal DVT, symptomatic nonfatal PE, or death from VTE through visit 3 or day 42.

b The associations of treatment and efficacy outcomes were found using logistic regression and stratified by dosing criteria (severe renal dysfunction, concomitant P-glycoprotein inhibitor, or neither).

c Interaction for D-dimer category.

d Interaction for D-dimer concentration.

**Fig. 1:**
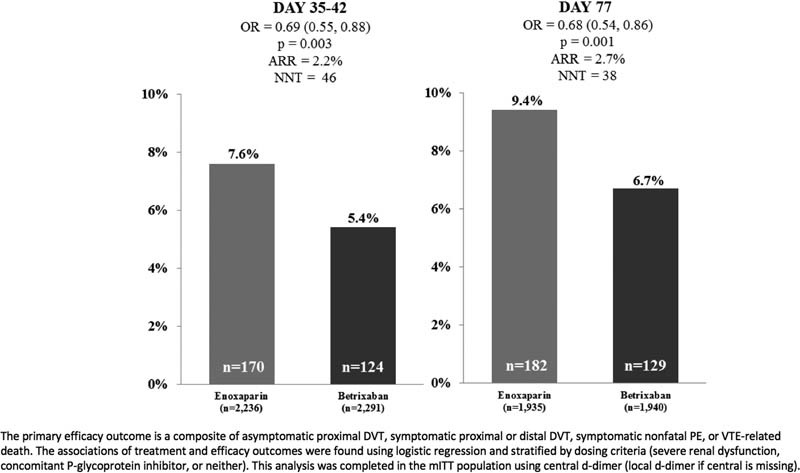
Primary efficacy outcome for D-dimer-positive patients through visit 3 and end of study.

When analyzed as a categorical variable, a positive D-dimer (≥ 2 × ULN; corresponding to ≥ 1.00 µg/mL) was associated with an increased VTE risk (6.5% [ *n* = 294] vs. 3.2% [ *n* = 93]; OR = 2.10 [95% CI: 1.66–2.67]; *p* < 0.001). After adjustment for age, duration of hospitalization, prior VTE, and previous thromboprophylaxis ≤ 96 hours, D-dimer remained an independent correlate of VTE events (OR = 2.03 [95% CI: 1.59–2.58]; *p* < 0.001; Table 2 ). Among positive D-dimer subjects (≥ 2 × ULN), extended-duration betrixaban reduced the risk of the primary efficacy outcome versus enoxaparin (5.4% [ *n* = 124] vs. 7.6% [ *n* = 170]; OR = 0.69 [95% CI: 0.55–0.88]; *p* = 0.003) through visit 3. The number of subjects needed to treat to prevent one VTE event among D-dimer positive subjects was 46 (absolute risk reduction [ARR] = 2.2%; Fig. 1 ). Among subjects with negative D-dimer (< 2 × ULN), there were numerically but not significantly fewer VTE events among subjects treated with betrixaban. There was no significant interaction between D-dimer category and the treatment effect ( *p*_int_ = 0.53; Table 3 ).

The primary efficacy outcome was also assessed through the end of study or day 77. Among subjects with D-dimer ≥ 2 × ULN (corresponding to ≥ 1.00 µg/mL), betrixaban significantly reduced the risk for VTE compared with enoxaparin (6.7% [ *n* = 129] vs. 9.4% [ *n* = 182]; OR = 0.68 [95% CI: 0.54–0.86]; *p* = 0.001; Fig. 1 ). The number needed to treat to prevent one VTE event through 77 days among D-dimer-positive subjects was 38 (ARR = 2.7%). Subjects who were D-dimer negative experienced numerically but not significantly fewer VTE events. There remained no significant interaction between D-dimer category and the treatment effect through the end of the study at 77 days ( *p*_int_ = 0.57; Table 4 ).

**Table 4:** Interaction for D-dimer category or D-dimer concentration and treatment effect through end of study

Outcome a	Enoxaparin	Betrixaban	Odds ratio (95% CI)	*p* -Value b	*p* -Value for interaction c	*p* -Value for interaction d
Primary efficacy outcome						
≥2 × ULN *n* / *N* (%)	182/1,935 (9.4)	129/1,940 (6.7)	0.68 (0.54–0.86)	**0.001**	0.57	0.76
<2 × ULN *n* / *N* (%)	54/1,319 (4.1)	41/1,260 (3.3)	0.79 (0.52–1.19)	0.26		

a The primary efficacy outcome is a composite of asymptomatic proximal DVT, symptomatic proximal or distal DVT, symptomatic nonfatal PE, or death from VTE through visit 3, but carried through end of study (day 77) for this analysis.

b The associations of treatment and efficacy outcomes were found using logistic regression and stratified by dosing criteria (severe renal dysfunction, concomitant P-glycoprotein inhibitor, or neither).

c Interaction for D-dimer status.

d Interaction for D-dimer concentration.

### D-dimer and Safety Outcomes

Among subjects in the safety population, D-dimer concentration was not significantly associated with either major bleeding or the composite of major or CRNM bleeding in either treatment arm.

## Discussion

Increasing baseline D-dimer concentration as a continuous variable was associated with an increased risk of VTE (asymptomatic DVT, symptomatic DVT, nonfatal PE, or VTE-related death). As a categorical variable, a positive baseline D-dimer (≥ 2 × ULN; corresponding to ≥ 1.00 µg/mL) was also associated with an increased risk of VTE in both univariate and multivariate analyses. There was no modulation of the treatment effect of betrixaban, however, as a function of D-dimer concentration. While the interaction term was negative, the event rates among D-dimer-positive patients were greater than D-dimer-negative patients, and the absolute risk reduction was numerically greater among D-dimer-positive patients. One would need to treat 46 D-dimer-positive patients to prevent one event at 35 to 42 days and 38 D-dimer-positive patients to prevent one event through the end of the study at 77 days.

**Fig. 2:**
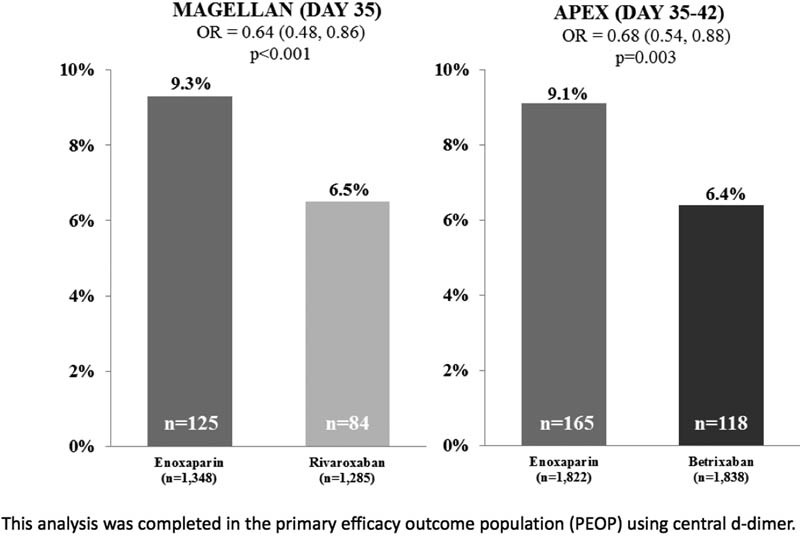
Primary efficacy outcome for D-dimer-positive patients through day 35 in APEX and MAGELLAN.

These results validate the hypothesis-generating observations in prior studies. 13 14 In the MAGELLAN trial, the incidence of VTE was 3.5 times greater for subjects with a central D-dimer ≥ 2 × ULN compared with those with a central D-dimer < 2 × ULN. 14 As central D-dimer increased in MAGELLAN, the absolute benefit of rivaroxaban over enoxaparin increased. 15 Among D-dimer-positive patients, there was a nearly identical reduction in VTE comparing active treatment to enoxaparin through days 35 to 42 or day 35 for both the APEX and MAGELLAN trials ( Fig. 2 ). Thus, the MAGELLAN trial could be viewed as hypothesis-generating and APEX as validating the increased absolute VTE risk reduction observed in D-dimer-positive patients. While the efficacy results were quite similar between MAGELLAN and APEX, with respect to safety in contrast, there was no excess major bleeding associated with extended-duration betrixaban, whereas there was excess major bleeding with extended-duration rivaroxaban.

To optimize VTE risk stratification among acute medically ill patients, D-dimer measurement can also be used in conjunction with risk assessment models. For instance, the IMPROVEDD VTE risk score (incorporating D-dimer into the IMPROVE score) has been demonstrated to improve the discrimination and reclassification of the IMPROVE model. 16 Adding D-dimer measurement to the IMPROVE score has also been implemented in the enrollment criteria of an ongoing trial for preventing hospital-associated VTE (Medically Ill Patient Assessment of Rivaroxaban Versus Placebo in Reducing Post-Discharge Venous Thrombo-Embolism Risk [MARINER]). 17

## Limitations

The cutoff for D-dimer was determined retrospectively, although the cutoff of ≥ 2 × ULN has been studied previously. Future prospective studies are required to validate the findings from this post hoc analysis. It is unknown if a D-dimer greater than 2 × ULN reflects the presence of clot at baseline, since no ultrasound was performed at baseline. The results obtained using central D-dimer resulting from a single quantitative test format may differ from the results obtained using local D-dimers, because different assays with distinct sensitivities and specificities were used at different sites. 18

Only a single D-dimer measurement was available at the time of enrollment, and no subsequent D-dimer measurement was available to determine if betrixaban reduced D-dimer concentrations greater than enoxaparin over time. Likewise, the association of temporal changes in D-dimer with clinical outcomes could not be evaluated. Whether extending VTE prophylaxis at discharge or at the end of parenteral therapy based on D-dimer concentration at that time would improve VTE risk is not known.

## Conclusion

Elevated baseline D-dimer is associated with a greater risk of VTE events, both as a continuous variable and as a discrete variable. Extended-duration betrixaban was superior to standard-duration enoxaparin, regardless of D-dimer concentration at baseline.
